# Beyond aesthetics: A case report of pneumosinus dilatans frontalis presenting with headache^☆^

**DOI:** 10.1016/j.ijscr.2024.109272

**Published:** 2024-01-13

**Authors:** Akbar Bayat, Sara S. Nabavizadeh, Tayebeh Kazemi

**Affiliations:** a Fellowship of Head and Neck Surgery, Private Farmaniyeh Hospital, Tehran, Iran; b Student Research Committee, Shiraz University of Medical Sciences, Shiraz, Iran; c Otolaryngology Research Center, Department of Otolaryngology, Shiraz University of Medical Sciences, Shiraz, Iran

**Keywords:** Pneumosinus dilatans, Frontal sinus hypertrophy, Craniofacial surgery, Frontal bossing

## Abstract

**Introduction and importance:**

The Pneumosinus Dilatans (PSD) Frontalis is an uncommon condition characterized by abnormal enlargement of the aerated frontal sinus with normal thickness sinus walls. The major complication is aesthetics; however, some cases present with sinus obstructive symptoms.

**Case presentation:**

A 32-year-old male presented with complaints of an asymmetrical protrusion on his forehead, as well as recurrent headaches. No signs of sinusitis were detected by periodic examination. Computed tomography demonstrated the presence of large frontal PSD. Due to aesthetic concerns, the patient was selected for forehead aesthetic surgery. The operation was performed through a bi-coronal incision to expose the supraorbital areas. The anterior wall of the right frontal sinus was removed, divided into 2 sections, and fixed into the proper location, and then the sinus outflow was widened. An asymmetric brow lift was then performed to correct the asymmetric brow position. Good results were attained, the patient's headache was resolved, and he was pleased with his appearance.

**Discussion:**

Although the most prevalent complaint of patients with PSD is aesthetic, some patients exhibit concomitant symptoms, including headaches and sinus obstruction. The constriction and partial obstruction of the sinus ostium may cause sinus cavity hypertrophy. Therefore, re-establishing sufficient drainage for the sinus by opening the sinus ostium is recommended during the reconstruction of the forehead's natural architecture to reduce headaches and recurrence of sinus hypertrophy.

**Conclusion:**

A combination of a bi-coronal approach regarding aesthetic surgery and sinus outflow widening achieves a desirable outcome that gives a good short-term follow-up result.

## 1 Introduction

The aesthetic dimensions of facial features, particularly the forehead, hold substantial significance in the broader context of our perception of attractiveness and overall facial harmony within the medical field (1). The prominence and characteristics of the supraorbital area are pivotal components contributing to the assessment of facial aesthetics, and deviations from the norm often pose unique challenges in clinical practice (2). Pneumosinus dilatans (PSD) is a rare pathological condition characterized by hyperpneumotization of one or more paranasal sinuses, resulting in deformation and displacement of the surrounding bony and soft tissues, leading to varying signs and symptoms (3). The frontal sinus seems to be the most affected paranasal sinus, followed by the sphenoid, maxillary, and ethmoid. In most patients, PSD frontalis is benign, and clinical symptoms are often attributed to the displacement of the structure. In situations with outward sinus extension, the most prevalent complaint is Cro-Magnon-like brows, which cause substantial cosmetic concerns (4). On the other hand, inward sinus expansion into the orbit, nose, and other sinuses is accompanied by more severe symptoms, including increased sinus pressure, headache, and diplopia (5).

Here, we present a case of PSD with asymmetrical bulging in the supraorbital region and orbital rim, leading to aesthetic and functional limitations for the patient, i.e., chronic headaches. Moreover, we highlight the advantages of re-establishing adequate drainage of the frontal ostium besides repairing the bony deformity. The study has been reported in line with the SCARE criteria (6).

## 2 Case presentation

A 32-year-old man was referred to the otolaryngology department in Farmanieh Hospital (Tehran, Iran), complaining of an asymmetrical protrusion at the supraorbital region. According to the patient, he first noticed the prominence of the frontal area at the age of 20, which continued to expand slowly until he was 25. The patient reported recurrent headaches localized to the forehead area, characterized by episodes lasting several hours (up to a day) and irresponsive to painkillers. These headaches occurred 2 to 4 times per week without specific triggering factors identified. Despite thorough examinations and investigations conducted by specialists to ascertain a diagnosis for migraine or other neurological disorders, an accurate and definitive diagnosis remained elusive. The patient's medical history did not include any instances of surgery or severe trauma to the craniofacial region, and there were no evident indicators of sinus infection, rhinitis, or allergies upon examination.

**Fig. 1:**
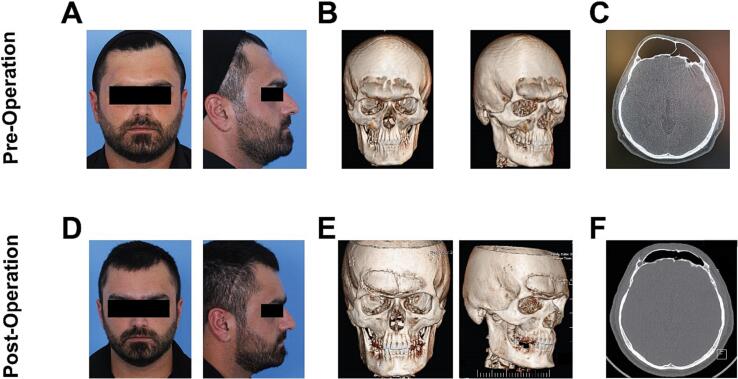
**(A)** Preoperative frontal and lateral views showing asymmetrical frontal bossing more sever in right side. **(B)** Preoperative computed tomography scan (3D frontal and right view), showing extensive pneumatization and expansion of the frontal sinuses. **(C)** Preoperative computed tomography scan of paranasal sinuses (lower axial view), showing expansion of the frontal sinus where the anterior table of the frontal sinus was pushed forward without any bony erosion. **(D)** Postoperative frontal and lateral views showing resolved frontal bossing and successful facial contouring. **(E)** Postoperative computed tomography scan (3D frontal and right view), showing proper bone reposition and frontal bossing correction. **(F)** Postoperative computed tomography scan of paranasal sinuses (lower axial view), showing normal frontal sinus structure without mucosal swelling.

Physical examinations revealed asymmetrical and more severe right-sided excessive protrusion of the frontal sinuses. The form and position of the brows were asymmetrical, and the right brow was depressed owing to the abnormal protrusion of the inferior sinus wall (Fig. 1A, B). The lower right orbital roof was responsible for the asymmetries seen in the eye shape. Both frontalis muscles were functional, although the right frontalis was somewhat weaker than the left when the eyebrows were raised. No soft tissue mass in the region of the forehead was felt. A nasal examination revealed no evidence of sinusitis or sinus discharge. There was no evidence of diplopia or blurred vision, and each eyesight was normal. The cause of the protrusion was unknown, and there were no functional problems. Laboratory results were within normal limits.

A computed tomography (CT) of the frontal sinuses showed a large pneumatized frontal sinus with no evidence of bony erosion or thinning of the sinus's walls (Fig. 1C). There was no evidence of sinus mucoceles or any mass in the surrounding area. Mild mucosal swelling was observed bilaterally at the opening of the frontal sinus, with the right side being more prominent than the other side.

Surgery was performed using a bi-coronal approach regarding the patient's aesthetic appearance. To do this, a coronal skin incision was made, and the forehead subgaleal flap was lifted. Through a transverse incision made approximately 1.5 cm above the superior orbital rim, the superiorly based peri-cranial flap and inferiorly based subperiosteal flap were elevated. Then, supraorbital nerve was identified and in order to preserve, the nerve was released from its canal at the superior rim of the orbit using an osteotome.

The periosteum was excised 1 cm above the orbital rim, and the superior-based pericranial flap was elevated. The periosteum was raised from the orbital rim and detached from the bone to a depth of 1.5 cm inside the orbit.

**Fig. 2:**
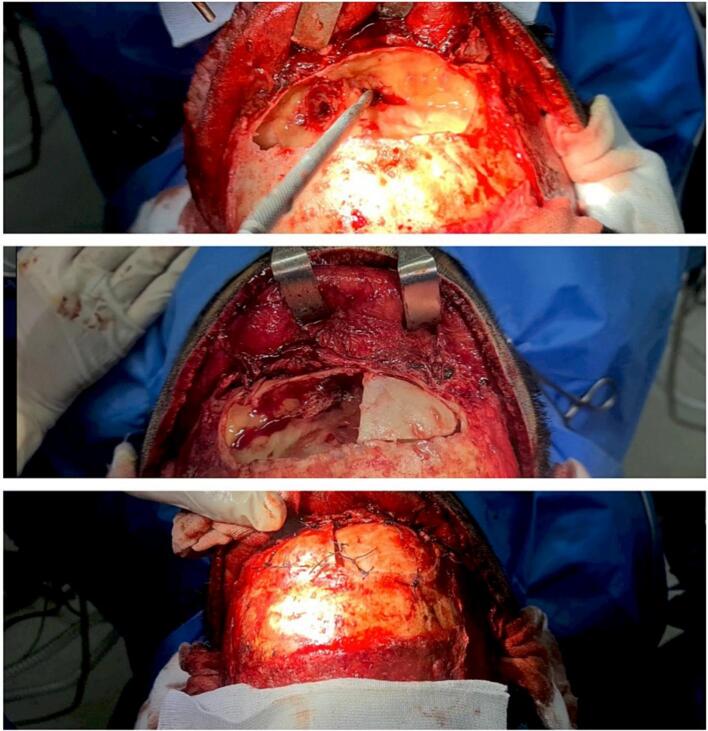
Intra-operative snapshots of Pneumosinus Dilatans Frontalis reconstruction surgery with a focus on maxillary sinus opening.

The anterior wall of the frontal sinus was then removed using a sagittal saw, with the blade positioned toward the center of the sinus to avoid damaging the sinus's surrounding walls. Mucosal thickening was observed on both sides of the frontal sinus floor. The narrow sinus ostium was widened bilaterally using a Curette and by mucosal preserving from the internal aspect toward the external. A small part of the septum between the two frontal sinuses was removed to provide easy access between the sinuses. The right superior orbital rim was released by osteotomy and fixed in a new higher position. Then, the anterior wall of the frontal sinus was shaved and contoured using a surgical burr; it was divided into two segments and then fixed in a new position using 0.1 Vicryl sutures. The pericranium was returned back and repaired. To overcome the asymmetry of the eyebrows, an asymmetric eyebrow lift was performed (more lifting on the right side) using 0.1 Vicryl sutures and fixed to bone tunnels. Subsequently, the coronal incision was repaired in two layers. No drainage was used (Fig. 2).

The patient was discharged immediately after regaining consciousness and stable vital signs (6 h after surgery). Cephalexin, topical ointment (Gentamicin), and acetaminophen were prescribed. In addition, the nasal wash serum was prescribed for daily application and was started the day after surgery. He was told to avoid blowing through his nose for two months.

After 6 months of follow-ups, good results were obtained; the patient had no complaints or sinus symptoms and was satisfied with his appearance (Fig. 1D). From his perspective, he did not anticipate the eyebrows to be more identical, and the outcome exceeded his expectations. In periodic examinations after the surgery, it was found that his headaches had fully subsided. There was some numbness in the upper part of the forehead after surgery that significantly reduced until six months after surgery. However, there was still some loss of feeling in that region, which the patient did not find annoying.

The three-dimensional reconstruction of the bones taken three months following surgery demonstrated the healing (Fig. 1E), and the CT scan revealed that the mucosal swelling of the sinus aperture had diminished (Fig. 1F).

## 3 Discussion

Pneumosinus dilatans (PSD) involves the expansion of an aerated sinus extending beyond normal wall boundaries without evidence of localized bone destruction, hyperostosis, or mucous membrane thickening (7). Until now, less than 150 cases have been diagnosed and reported in the literature; however, actual PSD prevalence is confounded by varying terminology used to describe the abnormal expansion of the sinuses. On the other hand, a significant proportion of patients only present with cosmetic concerns or are diagnosed on radiology alone; thus, it is safe to assume that the true incidence may be much higher than anticipated (8). Urken et al. adopted the most acceptable classification of hyperaeration of the paranasal sinuses in 1987, creating the current standard, and categorized it into three distinct subtypes based on the size of affected sinus and bony wall integrity: Hypersinus, Pneumocele, and PSD (9). A pneumocele is an aerated sinus extending beyond the sinus's normal anatomic boundaries with displaced sinus walls. Its invasive characteristics can differentiate it from PSD, leading to focal or generalized bony sinus wall thinning and erosion while hypersinus causes the increased size of a sinus cavity but within the normal range of the affected bone and with normal sinus walls (10).

There is a difference in PSD prevalence related to gender specificity, and it is considered that the occurrence in men is almost twice as common in women, with a peak incidence between 20 and 40 years of age (11). Furthermore, individual sinus involvement (80.1 %) was more common than multi-sinus involvement, in which the most affected sinuses are the frontal sinus (63 %), followed by sphenoid, maxillary, and ethmoid sinus, respectively (4,12).

The pathogenesis of PSD remains unclear, although various theories have been proposed that can be classified into primary and secondary types. Different hypotheses have been postulated for primary PSD, including a one-way valve, mucocele drainage, osteoclastic and osteoblastic activity dysfunction, hormonal dysregulation, and genetic predisposition (13). The secondary type of PSD is generally syndromic and is caused by either an alteration in the dynamics of cerebrospinal fluid (CSF) and decreased intracranial pressure, as in meningioma or arachnoid cyst, or by an overgrowth of sinuses to make up for association with brain agenesis, as in craniocerebral hemiatrophy (4,14,15). Among the aforementioned etiology of PSD, the one-way valve mechanism of the affected sinus cavity is the most accepted causative theory. An obstruction (such as a polyp, inflammatory mucosa, or tumor) along the sinus ostium may be linked with intermittent sinus outflow occlusion, increased sinus pressure, and consequent bone deformity (16). In addition to cosmetic issues regarding the bony deformity, obstruction of the frontal sinus outlet may lead to many sino-rhinal symptoms, including nasal obstruction, facial pain, and headaches (17,18).

Several therapeutic strategies have been suggested for the treatment of PSD-related craniofacial bone deformities, and myriad open procedures have been documented: remodeling and repair of the frontal bulge with alloplastic material with a high incidence of local complications; reduction of the anterior wall of the frontal sinus without obliteration of the sinus cavity with undesirable aesthetic consequences; removing the front wall of the frontal sinus, inverting it, and filling the resulted cavity with bone dust from the parietal region with the creation of an extra donor site; removing the anterior wall of the frontal sinus and replacing it with bone attached with mini plates or a titanium mesh plate (12,13,19). The most popular craniofacial reconstruction open surgery technique for individuals with frontal sinus involvement is a bi-coronal incision with osteotomy bone resurfacing of the outer table of the frontal bone (20).

Although patients with PSD are frequently referred due to primarily aesthetic concerns, it is imperative to recognize that a considerable number of patients present with concomitant or isolated symptoms of sinus obstruction that demand meticulous consideration. Hence, relying solely on facial bone reconstruction proves inadequate for individuals dealing with both functional and cosmetic manifestations. Berania et al. describe the conservative management of two PSD patients with minimal forehead bossing and progressive nasal obstructive symptoms (18). Furthermore, there are reports of endoscopic widening of the frontal sinus ostium to resolve obstructive symptoms and substantial bone deformities ([21], [22], [23]). Moreover, Patel et al. presented surgical management of the excessively distended frontal sinus lacking local defects depending on the patency of the nasofrontal duct, recommending endoscopic restoration of function in the absence of a cosmetic defect (8). However, it is crucial to underscore the nuanced roles and contextual relevance of both endoscopic and open surgical modalities in managing PSD-related craniofacial bone deformities. Endoscopic surgery stands as a pivotal strategy, especially when addressing functional concerns, such as sinus obstruction or mild bone deformities commonly associated with PSD. Its minimally invasive nature allows for targeted interventions aimed at widening the sinus ostium and ameliorating obstructive symptoms, thereby facilitating improved sinus drainage. Nevertheless, the utility of open surgical intervention becomes imperative when faced with intricate and extensive bone deformities. Open surgery provides unparalleled access and precision required to meticulously address complex structural anomalies prevalent in severe cases of PSD-related bony expansions. This approach enables meticulous manipulation and restoration of both form and function within the frontal sinus architecture. Furthermore, open surgical techniques encompass a comprehensive spectrum of interventions, encompassing reshaping of frontal bulges, realignment, and reinforcement of frontal sinus walls, while concurrently addressing aesthetic concerns alongside ensuring optimal functional outcomes. Therefore, while endoscopic procedures serve as an invaluable tool in select contexts, open surgery remains the cornerstone in managing cases characterized by substantial craniofacial bone deformities associated with PSD. (23).

Our findings suggest that when the nasofrontal duct is involved, surgical therapy for PSD frontalis aims not only to repair the bone distortion but also to restore a functional drainage system for the sinus. Restoring the frontal bone to its original position and mechanically widening the frontal sinuses outflow, along with routine nasal irrigation with saline solution, is an effective treatment strategy. Our findings further indicate that sino-rhinal symptoms in PSD seem to be directly connected to sinus drainage restriction due to edematous mucosa and should thus be treated directly.

Our study faces some limitations. The investigation presented in this case report is based on a single patient, restricting the generalizability of the findings. Additionally, the follow-up duration might not cover all potential long-term outcomes or recurrence rates of PSD post-surgical intervention. These limitations highlight the necessity for larger-scale studies with extended follow-up periods to validate the effectiveness and long-term outcomes of the proposed treatment approach.

Acknowledging the valuable input, we recognize the need for future investigations in the management of PSD. Our study provides a basis for further exploration. Subsequent research could delve into minimally invasive approaches and advanced surgical methods to enhance functional outcomes while considering aesthetic aspects. Moreover, elucidating the underlying pathophysiology of PSD might reveal potential targets for more precise therapeutic interventions.

## 4 Conclusion

In conclusion, our study has contributed significant insights into the multifaceted nature of PSD and its treatment strategies. Beyond highlighting the aesthetic implications, our findings emphasize the relevance of addressing functional symptoms related to sinus obstruction in PSD patients. Our study advocates for a comprehensive approach that involves not only correcting craniofacial bone defects but also restoring a functional drainage system, showcasing potential avenues for alleviating symptoms such as headache and facial fullness and mitigating the risk of relapse in PSD cases.

## Abbreviation

PSD: Pneumosinus dilatans
CSF: cerebrospinal fluid
CT: computed tomography

## Ethics statement

The studies involving human participants were reviewed and approved by this study were approved by the Ethics Committee of the Shiraz University of Medical Sciences (#18146–01–02-87). The patient has provided written informed consent to participate in the research and is authorized to publish the study. The patient/participant provided his written informed consent to participate in this study.

## Funding

There is no source of funding.

## Author contributions

AB contributed to the patient's surgery and review of the manuscript. SSN contributed to the literature review, review of the patient's information, and drafting of the manuscript. TK contributed to reviewing the manuscript and providing guidance on the approach to the topic. All authors contributed to the article equally and approved the submitted version.

## Guarantor

Tayebeh Kazemi.

## Declaration of competing interest

The authors declare that the research was conducted in the absence of any commercial or financial relationships that could be construed as a potential conflict of interest.

## Data Availability

The original contributions presented in the study are included in the article/Supplementary material, further inquiries can be directed to the corresponding author/s.
